# Seafood Choice and Consumption Behavior: Assessing the Willingness to Pay for an Edible Sea Urchin

**DOI:** 10.3390/foods12020418

**Published:** 2023-01-16

**Authors:** Alessandro Petrontino, Fabio Madau, Michel Frem, Vincenzo Fucilli, Rossella Bianchi, Adele Annarita Campobasso, Pietro Pulina, Francesco Bozzo

**Affiliations:** 1 Department of Soil, Plant and Food Sciences, University of Bari Aldo Moro, Via Amendola 165/A, 70126 Bari, Italy; 2 Sinagri s.r.l., Spin Off of the University of Bari-Aldo Moro, Via Amendola 165/A, 70126 Bari, Italy; 3 Department of Agricultural Sciences, University of Sassari, Viale Italia, 39A, 07100 Sassari, Italy; 4 Lebanese Agricultural Research Institute, El Roumieh Zone, Qleiat, Keserwan, Lebanon

**Keywords:** choice experiment, *Paracentrotus lividus*, seafood, sea urchins consumption

## Abstract

Consumers’ behavior towards sea urchin and preferences towards their origin certification and place of consumption may condition their market. In this context, the aim of this research was to elicit the preferences and perceptions of Italian sea urchin dishes using a discrete choice experiment (DCE) approach. A field survey of 453 respondents in Apulia (southern Italy) was conducted for this purpose. The DCE revealed that the origin certification of sea urchin provided Apulia’s consumers a high utility with a great pleasurable service in restaurants in which this species was served as a principal dish or seasoned with pasta or pizza. The DCE also showed that the consumption utility of sea urchin was related to a greater influence by place of purchase, place of consumption, technique of conservation, appearance, quality label, fishing zone, low price, male buyer, and, finally, medium and high incomes. Furthermore, Apulian consumers were willing to pay EUR 10.53/dish as an overall average for safe and certified sea urchin consumption. Given this, this research may promote the creation of a local sea urchin brand through the adoption of a market policy and a particular regulation related to the certification of origin, enhancing the competitiveness of this marine heritage species.

## 1. Introduction

### 1.1. Context and Importance

**Figure 1:**
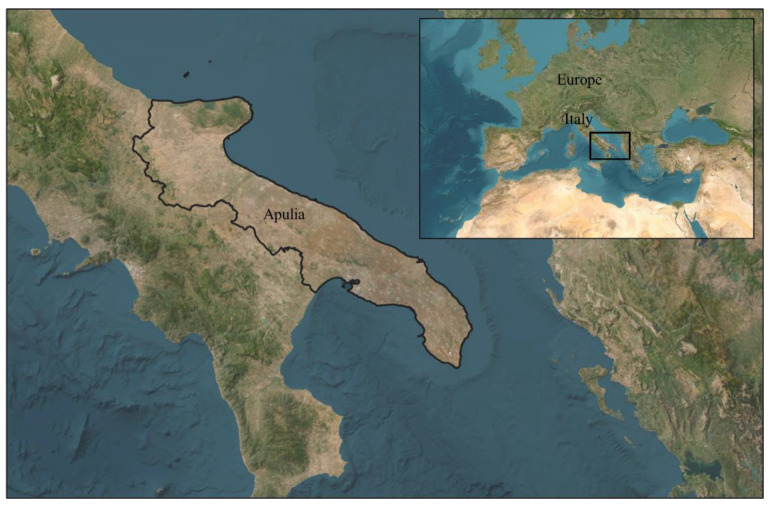
The European geographic location of the study area—Apulia region (southern Italy). Source: our elaboration.

Apulia (southern Italy, Figure 1) is a region that is deeply affected by the presence of the Adriatic Sea. This is also very evident in its typical cuisine and gastronomic restaurants, composed of traditional dishes, many of which are based on fish and seafood such as *Paracentrotus lividus* (Lamarck, 1816, Figure 2), an edible purple sea urchin [1], consumed mainly raw accompanied by bread and wine. This seafood species is very common throughout the Mediterranean Sea and the eastern Atlantic, from Scotland to the Canary Islands. Furthermore, this species holds a very important ecological role, as it helps to regulate the balance of the Mediterranean marine ecosystem [2]. According to the FAO database, the world harvest of sea urchin was estimated to be approximately 63 thousand tons in 2018 [3]. In the European market, Iceland, Spain, France, and Italy constitute the main producer countries. Nevertheless, the latter is one of the main European consumers of sea urchin, with approximately 2000 tons of product consumed every year. At the national level, the regions that are the most active in the collection of *Paracentrotus lividus* are Apulia, Sicily, and Sardinia [4]. Since sea urchin is a product with high commercial value, it is often subject to phenomena of unauthorized harvesting activity. It is important to highlight how Italy, with a production of approximately 107 tons a year, can no longer meet the growing domestic demand; thus, it is forced to import significant shares of sea urchin, especially during the summer season, from nations such as Canada (increase of 294% in recent years), Chile, or other European trading partners, including France and Spain. In addition, the value that the product assumes is strongly influenced by the state of conservation, the volume of imports, and the methods of processing and storage [3].

Users of sea urchin include not only the local inhabitants of the production areas but also tourists, whose demand has played a decisive role both in determining the increase in volumes and in the expanding periods and methods of consumption [5]. The taste of its gonads (the edible part) is appreciated all over the world [1]. Moreover, the semi-finished product obtained from the removal of the gonads, their packaging, and subsequent sterilization in glass containers [6] guarantees greater profitability than the sale of the hedgehog sold for fresh consumption.

### 1.2. Analogous Experimental Literature

Live sea urchins are considered the best quality product on the market. Their price is conditioned by different aspects: quality, origin, species, and organoleptic properties of the gonads. The latter are used for the preparation of numerous dishes, including spaghetti and/or pizzas with sea urchin, mainly when festivals and culinary events are organized according to Apulian popular traditions. When addressing these aspects, it is important to understand what drives consumers to purchase seafood such as sea urchin. In the last two decades, numerous studies have analyzed the factors that influence consumer preferences and calculated the willingness to pay (WTP) by types of seafood and for specific attributes [7,8,9]. Carlucci et al. [10] explored consumer behavior when buying fish and seafood products in the context of developed countries, highlighting an increasingly selective European consumer, even when it comes to seafood products. Regarding the key attributes of seafood purchasing decisions, a great number of studies have analyzed the importance of origin country, species type, presentation, size, seasonality, color, and price. The marketability of fish products and environmental and animal welfare awareness has been studied through labeling to specify the origin of sea products [11]. In countries such as the United Kingdom (UK), Japan, the United States of America (USA), and Germany, consumers are willing to pay a price premium to buy ecolabeled seafood, but the attributes “species” and “origin” tip the scales [11,12]. As such, both the literature and market behavior demonstrate how consumer interest in the origin of food, in general [13,14], and seafood, in particular, is gradually growing.

Moreover, other studies have underlined the importance of country-of-origin information in decisions on fish consumption [15,16,17]. Jaffry et al. [15] indicated that UK consumers preferred home-caught fish over imported fish. Similarly, Claret et al. [16] and Lawley et al. [17] highlighted that consumers preferred locally farmed species to imported equivalent species. Carlucci et al. [10] also confirmed that the country of origin complements ecolabeling as the most important attributes in purchasing and consumption decisions, and Hinkes and Schulze-Ehlers [18] concluded their study on pangasius and tilapia by stating that, overall, the country of origin together with sustainability are relevant factors influencing purchasing decisions, but aspects related to taste and preferences for some fish species could be even more significant in the German context of these seafood products imported from Bangladesh or Vietnam. However, other studies on consumer preferences that have analyzed both country of origin and sustainable production have shown that the country of origin has, on average, a greater effect on consumer preferences than sustainability [19,20].

In addition to the origin attributes, a few studies have investigated consumers’ perception of fish preferences in relation to attributes such as “presentation of the dish” and “place” where it is consumed. Moreover, a few studies have focused on the consumption behavior of sea urchin species. In fact, the purchase consumer behavior of salmon, as a seafood species, has been largely examined [21,22,23,24,25,26,27,28,29,30,31,32,33,34], followed by oysters [10,24,25,35,36,37,38], shrimps [24,25,39,40,41,42], pangasius [18,28,31,43], and tuna [9,28,44,45]. As such, all these studies are united by the adoption of product-specific attributes (Supplementary Materials Table S1) whereby consumers maximize their satisfaction utility and perceived product quality [46].

### 1.3. Purpose, Justification, and Significance

In this regard, within the project “TuGePlAl”, funded by the Apulia region (Appendix A), an economic study of sea urchin supply and demand was conducted to determine the main characteristics of its economic value chain, with particular attention to the analysis of the demand for fresh and processed marine products and the main sales channels, as well as the study of the supply, structure, and flows of the production chain in terms of volume and value. In this context, this research was carried out to elicit factors allowing to understand the behavior, preferences, and purchasing decisions of Italian consumers towards selected specific dishes based on seafood and to estimate their WTP for an edible local sea urchin, qualified as a niche product, caught from the Apulian Sea and sold in its markets. Precisely, this research addressed three interlinked research foci: (i) What are Apulian consumers’ behavior and propensity towards consuming sea urchin? (ii) What is their WTP for the presence of certification and the place of consumption of sea urchin? (iii) How do their socioeconomic characteristics influence their WTP? As such, this research contributes to the scientific literature in various dimensions. First, there is a dearth of studies that have applied a DCE to elicit sea urchin consumers’ behaviors and preferences as supported by the analogous experimental literature, described above (Section 1.2). Second, the Apulian seafood market has never been focused on consumer behavior, preferences, and WTP towards seafood and, particularly, sea urchin food. As such, referring to this region as a study area, we complement a previous study [47] conducted to propel Sardinian (an autonomous Italian region and the second-largest island in the Mediterranean Sea) consumers’ behavior and to elicit their WTP for sea urchin in varying dish patterns, including certification and place of consumption as particular attributes. In this framework, the study explored new insights into Italian regional differences in seafood preferences utility. Third, the current econometric valuation study is essential for the anticipation and setting up of market and innovation outreach strategies by seafood suppliers and restaurateurs that would meet Italian consumers’ expectations and, consequently, enhance their financial performances. Fourth, examining consumers preferences and perception has also public health implications in terms of increasing communication and information concerning origin labeling on seafood to impact the healthy consumption of seafood species such sea urchin. For these purposes, we used a discrete choice experiment (DCE) approach by means of a multinomial logit model (MNL) and mixed logit model (MXL), and the following section addresses how this was designed.

## 2. Materials and Methods

### 2.1. Discrete Choice Experiment

**Figure 3:**
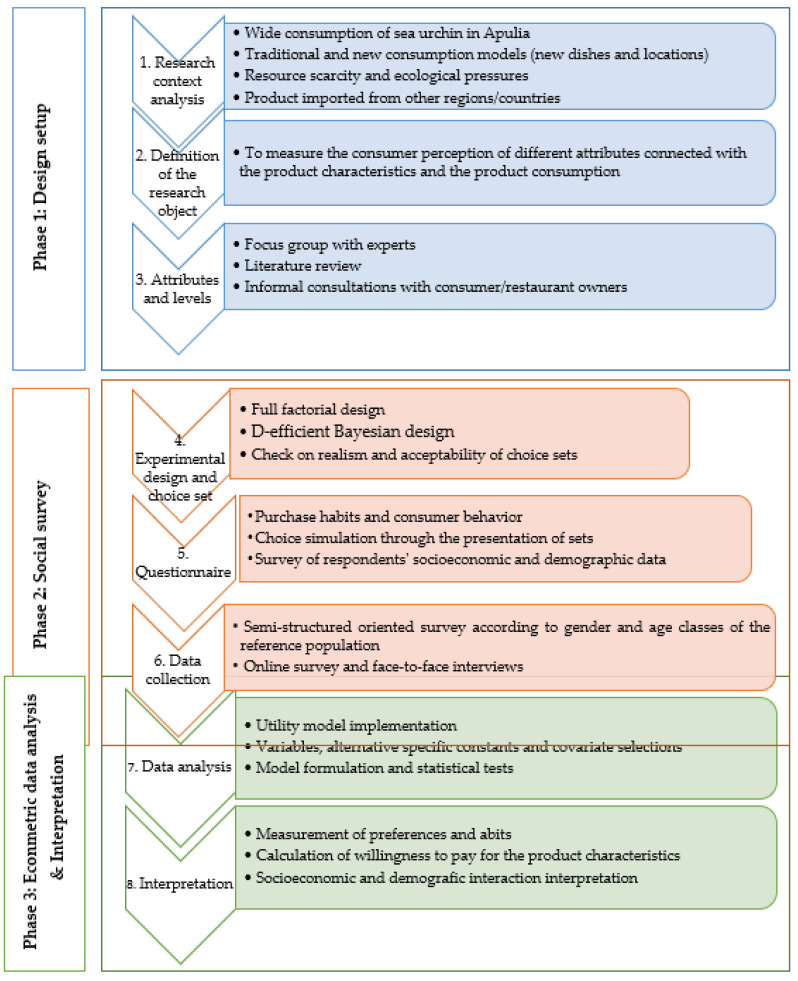
Overview of the discrete choice experiment (DCE) approach. The diagram highlights the steps in each phase of the DCE that were considered in our study, in which steps 1 and 2 have already been described in Section 1.

DCE, a well-established method, is widely used to understand consumers’ preferences and to estimate their WTP towards food product characteristics (i.e., attributes) such as food and/or seafood. Based on utility theory, we conducted the DCE in 3 phases which were divided in 8 steps (Figure 3), as follows: Phase 1—design set-up, which included the (i) research context analysis, (ii) definition of the research object, and (iii) selection of the attributes and the assignment of levels. Phase 2—survey, which included the (iv) experimental design and construction of choice sets, (v) realization of the questionnaire, and (vi) data collection. Phase 3—analysis and interpretation of the data, which included the (vii) data analysis using an econometric-based model and the (viii) interpretation of the preferences and the estimation of the WTP for the product characteristics.

### 2.2. Selection of Attributes and Assignment of Levels

We selected five attributes, inspired by Furesi et al. [47], as presented in Table 1. The selection of these attributes was justified by their importance in terms of traceability for Italian consumers’ WTP. In fact, we believe that the certification of a product, as a trustworthy attribute [42] and regarded also by consumers as a food safety attribute, is decisive in marketing strategies that may help to improve the performance of the sea urchin market. With respect to the place of consumption, we also considered that the significance of the consumption circumstances could influence or affect consumers’ WTP. Regarding the price, this attribute was considered here as a discrete variable [48], and its level was based on those applied above and below the current average retail selling price of the most popular Apulian seafood dishes.

### 2.3. Experimental Design and Choice Set

An important step in a DCE concerns the definition of the experimental design. In this regard, given the excessive number of alternatives (2^4^ × 4) resulting from the combination of the selected attributes and their respective levels (Table 1), we carried out a D-efficient Bayesian design, which allows for the maximization of the statistical efficiency by minimizing the D-error [49,50], using Equation (1). Starting from 2016, according to the possible alternative combinations, in addition to the “no choice” option, we generated 16 reasonable profiles arranged in one block [15] of 8 choice sets. The latter included 2 alternatives (Options A and B) and a “no-choice” (Option C), as presented in Table 2. The first four choice sets contained the main course alternatives. The other four included the starter alternatives. In each choice set, a sea urchin dish type was compared with a seafood dish type. We also supported each choice set using visual symbols [51] to facilitate the selection and to avoid consumers’ confusion. As such, the respondent selected the option that maximized their total utility regarding sea urchin consumption under a budget limitation [26,52].

(1) $$N=\frac{J^{n}\;\left(J^{n}-1\right)}{2}$$

where *J* is the number of levels, and *n* is the number of attributes.

### 2.4. Questionnaire Design

We designed a social choice questionnaire [48] in 3 parts (Appendix A). The first part investigated the behavior and propensity to buy and to consume sea urchin, along with 7 general categorical questions, using a Likert scale tool [53], which provides more information than binary answers, such as: “Have you ever bought sea urchin for domestic consumptions or during aperitifs/lunches/diners outside your home?” (Q1); “Where do you usually acquire sea urchin?” (Q2); “Where do you habitually consume sea urchin?” (Q3); “When purchasing sea urchin, how much attention (high, medium, or low) do you pay to the following characteristics: price, appearance, in terms of color and size, storage method, reliability of the seller, place of sale, presence of a label of origin and quality, information about the fishing area?” (Q4); “According to your personal experience, how much (high, medium, or low) of the following characteristics affect the price of sea urchin: appearance, in terms of color and size, storage method, reliability of the seller, place of sale, presence of a label of origin and quality, information about the fishing area?” (Q5); “Which of the following sentences identifies better your behavior in relation to the purchase: I am willing to buy a larger quantity if the price is low, I am willing to pay a higher price for a safe and certified product, I prefer the quality and price ratio without considering the origin of the product, Other?” (Q6); “Which sentence identifies better your behavior regarding the consumption of sea urchin: I prefer to consume sea urchin at home, in my comfort zone; I prefer to consume sea urchin outside home, in a convivial context; I prefer to consume sea urchin in a formal environment?”. The second part of the questionnaire presented 8 purchase simulations (i.e., set choice) in which each respondent had to select between 2 options (A and B) among each set choice that differed in the type of dish, certified origin, place of consumption, and price. The set choice also included a no-purchase option (C). Table 2 illustrates an example of a set choice used in this study, displayed using images with a description in the caption [54]. In the third part of the questionnaire, we collected socioeconomic data on the respondents in relation to gender, age, residence, civil status, and family composition, level of education, work position, work sector, and annual household income. Our intention was to understand the potential significance of this kind of information on consumption behavior and preferences as well as on WTP levels. As such, we attentively pre-examined this questionnaire survey through a pilot social survey of 22 respondents.

### 2.5. Sampling of Respondents and Data Collection

We collected data from November 2021 till September 2022 through online and face-to-face surveys in several locations of the Apulia region, involving 453 valid seafood respondents, considering both the Apulian population’s age and gender distribution (Table 3), in which the sample presented a similar structure as the Apulian population. As such, we based our sampling on the Istat database [55], and we obtained an appropriate sample size of at least 385 Apulian respondents by using the calculator [56] within a margin of error of 5%, a confidence level of 95%, and a total population size of 3,295,839 inhabitants, in which age classes younger or equal to 18 years were not included in the formula of the sample size, as follows:

n = N × X/(X + N − 1)(2)

where X = Zα/2 2 × p × (1 − p)/(MOE)2 Zα/2 is the critical value of the normal distribution at α/2 (for a confidence level of 95%, α is 0.05, and the critical value is 1.96); MOE is the margin of error; p is the sample proportion; and N is the population size.

### 2.6. Econometric Model: Data Analysis and Interpretation

We based our econometric data analysis and interpretation, using NLogit version 5.0, on random utility models [46] in which the respondent based their purchase decision by choosing the option (A, B, or C, as mentioned above) that maximized their satisfaction utility and perceived seafood species quality. In other words, considering respondent *i*, who chooses the alternative that can guarantee the greatest utility among the *J* possible alternatives at a given occasion of choice *T*, the utility function is thus given by the following equation [57]:

(3) $$U_{ijt}=V_{ijt}+e_{ijt},\;i=1,\ldots ,I;\;j=1,\ldots ,J;\;t=1,\ldots ,T$$

where *V_ijt_* is the deterministic component, and *e_ijt_* is the random component, independently and identically distributed (IID).

Considering the individual characteristics to stress the heterogeneity of the preferences and assuming a linear utility function in the parameters for the deterministic component, Expression (3) can be reformulated as:

(4) $$U_{ijt}={\beta^{\prime }}_{i}x_{ijt}+\varepsilon_{ijt},\;i=1,\ldots ,I;\;j=1,\ldots ,J;\;t=1,\ldots ,T$$

where *β_i_* is a vector of *K* × 1 parameters to be estimated and inherent in utility, corresponding to *K* choice characteristics; *x_ijt_* is the *K* × 1 vector of the characteristics of choice concerning alternative *j* at the choice occasion *t* made by individual *i*.

In this regard, it should be noted that respondents may exhibit similar attitudes in their choices across choice sets, leading to the phenomenon of correlation and, thus, violation of the IID assumption. Expression (4), on the other hand, involves the introduction of a vector of *β_i_* parameters that are specific to the respondents and following a distribution *g*(*β*|*θ*), whose vector *θ* indicates the mean and variance. This specification, allowing for the above assumption to be relaxed, refers to the random parameter logit model (RPLM), which allows for the capture of the heterogeneity related to factors not observed but common to groups of respondents and able to influence their behavior, hence, decision making. The conditional probability on parameters *β_i_* that an individual *i* will choose a sequence of choices *s_i_* = {*s*_*i*1_, *s*_*i*2_,…,*s*_*iT*_}, given profiles *x_i_* = {*x*_*i*1_, *x*_*i*2_,…,*x*_*iT*_}, is given by:

(5) $$P\left(s_{i}|x_{i},\beta \right)={∐}_{t=1}^{T}\left[\frac{exp({\beta^{\prime }}_{i}x_{is_{it}t}}{\sum_{j=1}^{J}exp\left({\beta^{\prime }}_{i}x_{ijt}\right)}\right]$$

Integrating (5) with respect to the distribution of *β* yields the unconditional probability, so that:

(6) $$P\left(s_{i}|x_{i},\theta \right)=\overset{\;}{\underset{\beta }{\int }}P\left(s_{i}|x_{i},\beta \right)g\left(\beta |\theta \right)d\beta$$

However, (6) does not present a closed-form solution, so for the estimation of the model, simulated maximum likelihood methods are used [57,58]. Since Halton extractions are an efficient alternative to random extractions, in this study, the method was used of Halton at 200 extractions. In addition, a normal distribution was used for the functional form of the parameter density functions [59]. As regards the calculation of the WTP, that is, the contribution that respondents are willing to pay for each proposed product characteristic, the following expression was used:

(7) $$WTP_{A}=-\frac{\beta_{A}}{\beta_{P}}$$

where *WTP_A_* is the willingness to pay for attribute *A*; $\beta_{A}$ and $\beta_{P}$ are the estimated coefficients related to each attribute and price, respectively.

## 3. Results

### 3.1. Basic Descriptive Statistics

This section includes basic statistical results from the Parts 1 and 3 of the questionnaire, dealing with consumers’ general attitudes towards the behavior and propensity to consume sea urchin and their sociodemographic and economic characteristics in the studied area. Fish shops were the most popular places of purchase for sea urchin (42%), followed by restaurants (21%) and fishermen (15%). The majority of the respondents that purchased at fish shops consumed this seafood species at home (77.10%), while 18.70% of them consumed it in restaurants. Home was the place of consumption preferred by the majority of people purchasing at fishermen (49%). People that consumed urchins directly from the sea, purchased it from fishermen or caught sea urchin by themselves as hobby fishermen (Table 4).

Although 74% of the respondents in this social survey accorded a high level of attention towards the reliability of the seller, which means that their purchase decision was really influenced by this product feature in the studied area, followed by the relative importance of the aspect, mode of conservation, and purchase site of sea urchin (Table 5). With regard to their self-level experience with the level of influence of sea urchin features on its purchase price, very few respondents (2%) were convinced that the aspect of the product influenced its purchase price, but 52% of them confirmed the high level of influence of the origin and quality label (Table 6).

In general, sea urchin obtained the highest willingness to pay a price premium if the product was safe and certified (approximately 57%), followed in succession by its other features, such as an adequate quality/price ratio, without caring about the origin (32.3%), and price (approximately 11% of the respondents were willing to buy a larger amount if the price was low), as shown in Table 7. Furthermore, most Apulia consumers (57.17%) preferred to consume sea urchin outside, in a convivial context, while few of them (approximately 5%) preferred to consume this marine species in a formal context, as shown in Table 8.

Regarding the socioeconomic profiles (Table 9), on average, the respondents were middle-aged (48.51 years old), mainly residents of the Apulia region (94%), and equally divided between genders, since 49.2% were female, but they were widely differentiated with respect to family size (one member: 11.7%, two members: 25.6%, three members: 30.5%, four members: 25.6%, and more than four members: 6.6%). In terms of education, three major classes were observed: one with a high school diploma (approximately 41.3%), one with a bachelor’s degree (32.5%), and one with a post-bachelor’s degree (25.6%). Most of the respondents were employed (35.5%), and the total annual household revenue was distributed as follows: 28.70% (under EUR 20,000), 58.06% (EUR 20,000–40,000), 11.92% (EUR 40,000–60,000), and 1.32% (over EUR 60,000).

### 3.2. Econometric Results

#### 3.2.1. Sea Urchin Consumers’ Perception

The multinomial logit model (MNL) estimates are reported in Table 10, in which all coefficients of the concerned attributes presented positive signs, except for price, as envisaged, and were highly significant at the notable level of 1%. On the contrary, the ASC No choice coefficient (i.e., no buy) was equal to −0.14 and not significant. This result indicates that the respondents strongly preferred to consume dishes based on sea urchin presented as the main dish or starter. Further, the certificate of origin coefficient (“certification”), as anticipated, was positive and highly significant (ρ = 0). This finding denotes that “certification” provided consumers with high assurance or trust regarding, at least, the quality of the seafood species. In addition, the sign for the attribute regarding the place of consumption (“restaurant”) was positive and highly significant (ρ = 0), indicating that this attribute provided Apulian consumers with higher pleasurable service in restaurants in which sea urchin is served as the principal dish or seasoned/savored with pasta or pizza.

With respect to the RPL model, the log likelihood function and pseudo R^2^ fulfilled better values compared to the MNL model (Table 11). The RPL estimates (Table 12) show that all the concerned attributes were coherent with the MNL model, indicating also a high statistical significance at the 1% level and rejecting the hypothesis of homogeneous consumer preferences for sea urchin preparation and consumption. In other words, this result assumes variation in the utility coefficients across the respondents. The ASC No choice coefficient (i.e., no buy) was positive but not significant, indicating that consumers would gain utility from Option A or Option B over Option C. Moreover, the coefficient “certification” attribute was positive and highly significant as well as for the place of consumption “restaurant”, suggesting that consumers obtained high utility from a certified sea urchin product consumed in restaurants. Moreover, the interaction between ASC Opt-out, propensity variables, and the socioeconomic characteristics of the respondents was elaborated to explain this consumers’ heterogeneity in preferences for sea urchin. The findings reveal that the correspondent coefficients with negative and significant levels (Table 12) imply that respondents attributed a high degree of importance when making choices between options. In other words, the consumption utility of sea urchin was related to a greater influence by place of purchase “street vendor and mall”, place of consumption “sea”, “technique of conservation”, “appearance”, “quality label”, “fishing zone”, “more quantity for low price”, “male buyer”, and “medium and high incomes”. On the contrary, the coefficients with positive signs indicate that the correspondent covariates influenced Option C (i.e., No buy).

#### 3.2.2. Sea Urchin Consumers’ Willingness to Pay

**Figure 4:**
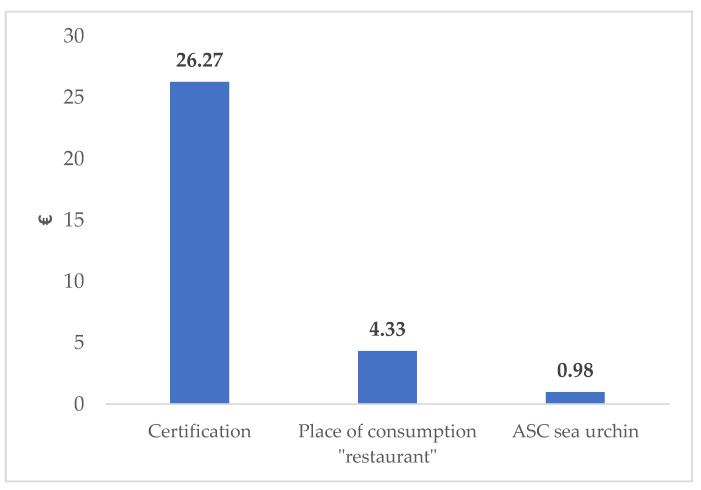
Apulian consumers’ willingness to pay for the consumption of sea urchin. ASC refers to alternative specific constant, which here represents the “none” option in each choice set.

The distribution of WTP for the selected attributed are presented in Figure 4, in which the highest WTP is determined by the “Certification” attribute (average € 26.27/dish), followed by the place of consumption “Restaurant” (average €4.33/dish), and ASC sea urchin (average €0.98/dish). As such, Apulian consumers are willing to pay € 10.53/dish on overall average for sea urchin consumption.

According to the random parameter logit model results, the purchase behavior of the respondents was influenced by some of the covariates included in the model. The characteristics of the respondents that had significate reflection on the willingness to pay were those contributing to the heterogeneity in the mean variation. The covariates that negatively affected the WTP were those with a negative sign, as shown by results concerning people who purchase from street vendors, who consume directly from the sea, and who pay attention to the product’s conservation. Moreover, the negative variation of the WTP was also constituted by those who think that price is influenced by the quality label and by the fishing zone. The same can be said for male buyer and households of medium- and high-income levels. The respondents’ characteristics with a positive reflection on the WTP were those that had a positive sign related to the interaction with the opt-out parameter, such as those who purchase from kiosks and fish shops and who pay attention to price, product appearance, and seller reliability. Moreover, a positive WTP effect could be observed for the respondents who thought that the place of purchase influences the price level and who are willing to pay more for a certified product.

## 4. Discussion

### 4.1. Interpretation and Comparison

The obtained WTP was relatively high (on average, EUR 27.3) for certification features, suggesting consumers’ perception and interest in greater and clearer labeling or information regarding the origin and source of the seafood product they consume. As such, the origin of the product appears here to be a determinant criterion for Italian consumers. This certainly reflects their concern regarding the sustainable conditions of harvesting and processing, as well as antifraud issues related to seafood products. In other words, information concerning this feature seems to prevail in the dish pattern presentation and/or the situational factors consumption attributes [60]. This result is consistent with Nguyen et al. [44], who found that improving communication on seafood species, preparation methods, and product forms prior to cooking was barely significant with respect to product origin, production methods, and sustainability certification. Moreover, our results are consistent with several other previous studies that estimated consumers’ WTP for sustainable seafood products through the perception of specific attributes [9,21,31,61,62,63,64,65], particularly the study by Bastounis et al. [66], in which consumers were willing to pay more for ecosustainable products and where ecolabels were a promising strategy to encourage this type of purchase.

Moreover, Carlucci et al. [10] found that, in Italy, consumers are willing to pay a significant price premium for certification labels but without preferring any kind of certification. In addition, our findings are in line with Risius et al. [67], who found that there was a clear preference for domestic seafood products, perceived as superior in quality, safety, and freshness compared to imported fish. Furthermore, Risius et al. [67], aiming to conduct a segmentation of fish consumers based on preferences for different product attributes to identify target groups for fish from sustainable aquaculture and for different countries of origin, identified five target groups, the most relevant (39%) of which gave the highest importance to the country of origin, while another (20%) gave priority to the country of origin over all other attributes. In addition, Nguyen et al. [44] showed that consumers perceive domestic seafood as safer and have a high willingness to pay for seafood of domestic origin. Chen et al. [36] analyzed Hawaiian chefs’ preferences for the attributes of locally raised oysters to calculate the marginal willingness to pay for this seafood. The study in question, therefore, addressed the attributes related to price, breeding site, freshness, and stability of supply. The results showed that the respondents favored fresh (and nonfrozen) oysters, locally raised (not imported from abroad) and supplied on the basis of availability (not according to a pre-established schedule). In addition, chefs were willing to pay USD 5.25 more for a dozen locally raised oysters; therefore, it might be interesting to delve into this research topic for other seafood and a larger sample of respondents (of 27) and from other countries. On the contrary, our results are not in line with Cantillo et al. [65], who analyzed and identified the main determinants of the frequency of fishery and aquaculture products (FAPs) for domestic consumption. Their results indicated that the main reasons for consuming FAPs were related to their healthiness, taste, and relatively low price. However, they also highlighted that the reason why consumers should consume them less frequently was related to a lack of understanding of the information that accompanies these products.

In relation to the place of consumption, recently, Nguyen et al. [44] published two different studies that revolve around the issue of the information presented on restaurant menus, the first of which delves into the preferences of American consumers in reference to obtaining information on seafood appetizers served in casual (midrange) and fine (upscale) restaurants. According to this research, the demand for information on the fish served varied according to the attribute submitted to consumers; however, more than 80% of respondents said they were satisfied with the species identification, cooking method, and price attributes.

### 4.2. Findings and Implications

The obtained results induce here public and private implications. In fact, examining consumers’ preferences according to some purchase attributes may urge cohesion and dialogue between fisherman practices and consumer behavior for a better protection of the biodiversity of marine ecosystems and, above all, species of sea urchin currently at risk of extinction in the study area. Furthermore, within the broader vision of the Europe 2020 strategy for smart, sustainable, and inclusive growth, this research implies innovative scientific and evaluative economic results/contributions in line with the EU’s priority regarding the promotion of environmentally sustainable resource-efficient fisheries that are innovative, competitive, and knowledge based. Overfishing of natural stocks of seafood such sea urchin to satisfy the growth of its demand, as well as the marine habitat damage, may generate a regional crisis in the production of this kind of sea product. To avoid this potential crisis, we need sustainable and innovative systems for the management of sea urchin production and consumption that will comprehensively attenuate the impact on natural sea resources. Given this, with the examination of consumption and potentially changing habits, it is possible to condition the market and reverse the course by supporting sustainable fishing and preserving the sea in the study area. With respect to the private implications, in addition to increasing consumers’ awareness and enhancing their nutrition–health conditions, this study may promote the creation of a local (Apulia) sea urchin brand through the adoption of a market policy and a particular regulation related to the certification of origin, recognizing that Apulian consumers are willing to pay a price premium for a safe and certified marine heritage. While respecting the complex balance of the marine ecosystems, the creation of such a specific geographic brand may allow for the achievement of good business performances and potentially increase the levels of competitiveness and profitability of fish stakeholders.

### 4.3. Limitations and Future Research Directions

Our study involved representative respondents considered as the “general public”, which may be considered relatively less valuable for some fish stakeholders such as seafood restaurateurs. Since this study revealed a respondent utility of consumption of sea urchin at restaurants, we suggest an extension on examining Italian consumer needs regarding sea urchin served at midscale and upscale restaurants [65] in Apulia and other southern regions of the country through a face-to-face field social survey and including another seafood species [44]. The use of an online survey was also a limitation of this study, since this kind of data collection may mainly exclude elderly persons [68,69]. As such, we may allow for extending the analysis of the consumer differentiation between communities and different age classes. Further, the sustainability of seafood production and consumption has emerged. Therefore, the list of the attributes may be extended in a further study to cover the impact of the environmental conditions of fishing and processing, nutritional/health claims, and safety information issues [70] on Apulia sea urchin consumers’ behavior and their WTP. Moreover, to avoid extreme values of the WTP, a payment card question could be suggested to respondents asking them: “if the price of a seafood dish (such as sea urchin) in a restaurant is EUR ‘value X’, how much more are you willing to pay as a % of this price for the selected attributes [44]”.

## 5. Conclusions

The analysis conducted revealed, among others, a particularly significant element with respect to the effects on the market of the fish product in question. The consumer was willing to pay a price premium towards the guarantee of purchasing a certified product, in terms of the quality and origin, and that was traceable in relation to all of the links in its value chain, organoleptically intact, and preferably of local origin. Meanwhile, this seafood attribute was linked to the contents of ecological–environmental sustainability on which the agrifood and structural and rural development policies of the European Union, as well as those of the United Nations, have been based since the end of the 1980s and which have led to the adoption of a Code of Conduct for Responsible Fishing [71]. Fishing, as well as aquaculture, represents crucial productive dimensions of socioeconomic systems, with a fundamental value in terms of ecological–environmental resilience. However, overfishing of certain species of sea urchin, as well as uncontrolled harvesting by unauthorized fishermen, has led to the application of specific management policies (i.e., catch quotas, rotational fishing, and aquaculture) to allow for the restoration of stocks and/or mitigation of the impact on natural populations. From this perspective, Apulia, with its 830 km of coastline along the Adriatic and, in part, along the Ionian Sea, can certainly express a significant determination in favor of the enhancement of small coastal communities and their socioproductive commitment within this value chain. Furthermore, classified as a problematic region of southern Europe, Apulia can certainly seize opportunities to activate structural development paths that hoard productive specificities, such as that, for example, of the *Paracentrotus lividus*, launching paths of cultural enhancement [72]. From this perspective, the results provide some suggestions concerning the direction in which to drive sea urchin enhancement policies, based on consumer preferences. In this sense—according to the high estimated value of the specific attribute—the potential positive role that the certification of regional origin of the product can assume in the added value creation is evident. More generally, the certification of origin or that certifying compliance with good catching and/or traceability practices along the supply chain (i.e., “from the sea to the table”) could represent, even more than the place of consumption, the main leverage to enhance such an important resource that, however, is at risk of overexploitation. Obviously, these results need to be corroborated by other studies and empirical evidence, if anything, by also evaluating any other attributes referable to sea urchins and not contemplated in this study or in other reference markets other than the Apulian one.

## Figures and Tables

**Figure 2:**
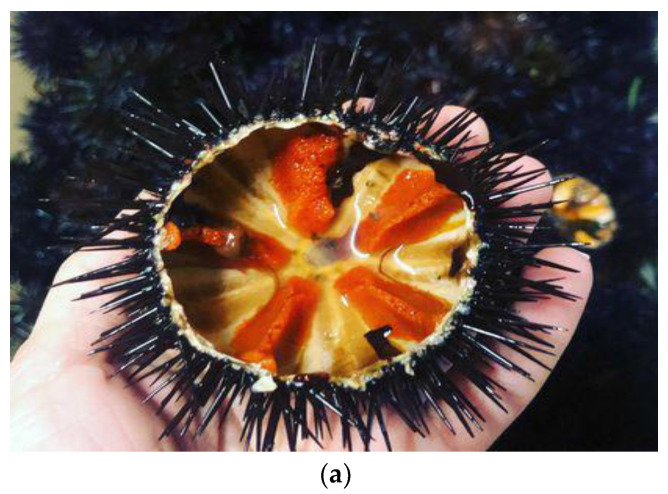

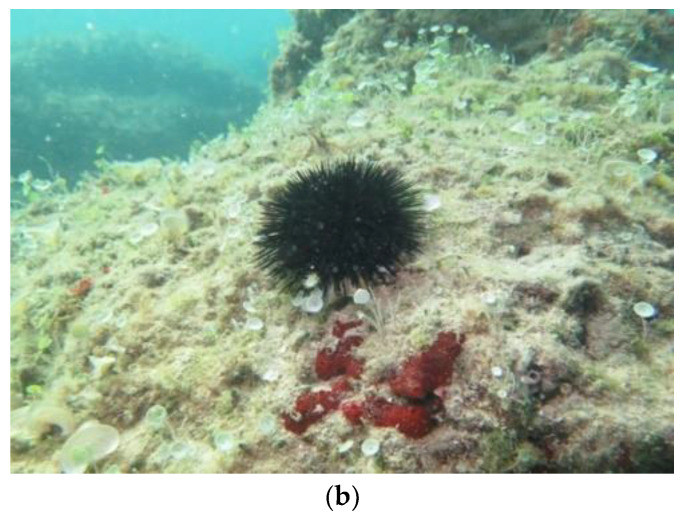
Photos of sea urchin from the Apulia region (southern Italy). *Paracentrotus lividus* (**a**) is also commonly referred to as the “edible female hedgehog”. The “male” or “black” hedgehogs (**b**) are none other than species of *Arbacia Lixula*. In Puglia, although the same applies to many other Mediterranean countries and to the parts of Spain and France bordering the Atlantic, the hedgehog that is harvested and consumed is the *Paracentrotus Lividus*, otherwise called “purple hedgehog” for the widespread presence, in its populations, of specimens whose spines show, precisely, a color tending to violet. Source: photos provided by Meliadò Eleonora, 2021, project “TuGePlAl” funded by the Apulia region (Appendix A).

**Table 1:** Attributes and levels used to elicit Italian sea urchin consumers’ preferences in the survey.

Attribute	Level Number	Level
Food type	2	1. Sea urchin 2. Seafood
Certification of origin	2	1. Yes 2. No
Place of consumption	2	1. Restaurant 2. Home
Dish type	2	1. Raw sea urchin as starter 2. Sea urchin with pasta as a main course
Price	4	1. EUR 10 2. EUR 15 3. EUR 20 4. EUR 25

**Table 2:** Example of a set choice used in the survey.

Choice Set in Which Raw Sea Urchin is Served as the Main Course		Choice Set in Which Raw Sea Urchin is Served as the Starter	
A	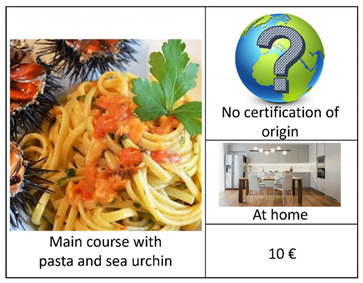	A	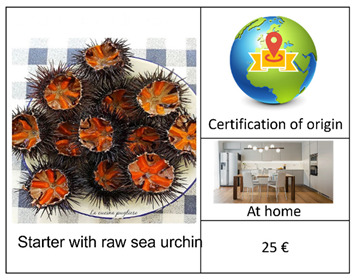
B	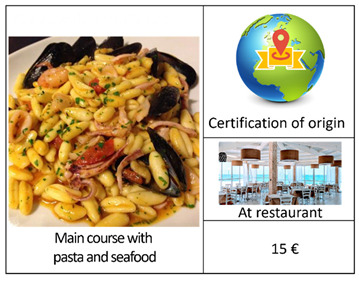	B	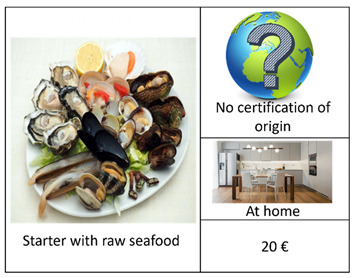
C	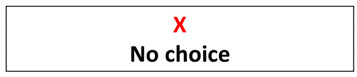	C	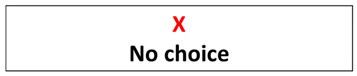
I will opt for: (Select one option using the symbol 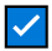 ) Option A: ------; Option B: -------; Option C:---------.		I will opt for: (Select one option using the symbol 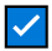 ) Option A: ------; Option B: -------; Option C:---------.	

**Table 3:** Sampling of the respondents taking into consideration the age, gender, and household revenue of the population in the Apulia region.

		Apulia Region	Sample
Age class	19–30	17.00%	13.00%
	31–50	31.00%	36.00%
	Over 50	52.00%	50.00%
Gender	Male	49.00%	51.00%
	Female	51.00%	49.00%
Annual household income (EUR)	<20,000 20,000–40,000 40,000–60,000 >60,000	31,156 (average)	29.00% 58.00% 12.00% 1.00%

Source: Istat, 2021 [55].

**Table 4:** Purchase sites by consumption place of sea urchin (in % of respondents), excluding restaurants (conventional restaurant and restaurant-fish, representing together more than 30%).

Place of Purchase		Place of Consumption (in %)				
	Total	Home	Restaurant	Fish Shop Restaurant	Sea	No Consumption
Online	0.00	0.00	0.00	0.00	0.00	0.00
Peddler	4.00	100.00	0.00	0.00	0.00	0.00
Shopping mall	1.00	100.00	0.00	0.00	0.00	0.00
Kiosk	1.00	0.00	0.00	100.00	0.00	0.00
Fisherman (direct sales)	15.00	49.00	4.00	18.00	29.00	0.00
Fish shop	42.00	77.00	19.00	4.00	0.00	0.00
No purchase	5.00	0.00	17.00	0.00	44.00	39.00

**Table 5:** Self-level attention on the characteristics of sea urchin among Apulian consumers (in % of respondents).

Sea Urchin Feature	Level of Attention (in %)					
	Low		Medium		High	
	Number	%	Number	%	Number	%
Price	60	13.00	242	53.00	151	33.00
Aspect	13	3.00	146	32.00	294	65.00
Mode of conservation	43	10.00	110	24.00	300	66.00
Reliability of the seller	20	4.00	100	22.00	333	74.00
Purchase site	28	6.00	152	34.00	273	60.00
Presence of origin and quality label	221	49.00	137	30.00	95	21.00
Information on the fishing zone	167	37.00	140	31.00	146	32.00

**Table 6:** Self-level experience with the level of influence of sea urchin features on its purchase price (in % of respondents).

Sea Urchin Feature	Level of Influence (in %)					
	Low		Medium		High	
	Number	%	Number	%	Number	%
Aspect	9	2.00	116	26.00	328	72.00
Mode of conservation	63	14.00	165	36.00	225	50.00
Reliability of the seller	94	21.00	115	25.00	244	54.00
Purchase site	56	12.00	97	22.00	300	66.00
Presence of origin and quality label	118	26.00	98	22.00	237	52.00
Information on the fishing zone	108	24.00	144	32.00	201	44.00

**Table 7:** Apulian consumers’ behavior related to sea urchin purchasing.

	Number	%
I am willing to buy a larger amount if the price is low	49	11.00
I am willing to pay a price premium if the product is safe and certified	258	57.00
I prefer an adequate quality/price ratio, without caring about the origin of the product	146	32.00
No answer	0	0.00
Total	453	100.00

**Table 8:** Apulia consumers’ behavior related to sea urchin consumption.

	Number	%
I prefer to consume sea urchin at home	144	32.00
I prefer to consume sea urchin outside, in a convivial context	259	57.00
I prefer to consume sea urchin in a formal context	20	4.00
Other (I prefer to consume sea urchin at the seaside)	21	5.00
No answer	9	2.00
Total	453	100.00

**Table 9:** Socioeconomic characteristics of the respondents.

Social and Demographic Variable (Unit)	Category	Median	Mean
Age (year)	-	50	48.51
		Number	%
Gender	Female	223	49.00
	Male	230	51.00
Residence	Inside Apulia region	426	94.00
	Outside Apulia region	27	6.00
Household composition (number)	1	53	12.00
	2	116	26.00
	3	138	30.00
	4	116	26.00
	5	30	7.00
Education level	Illiterate	0	0.00
	Elementary	0	0.00
	Lower secondary school	3	0.70
	Higher secondary school	187	41.00
	Bachelor	147	32.00
	Master and PhD	116	26.00
Work position	Student	7	1.00
	Unemployed	21	5.00
	Employed	161	35.00
	Entrepreneur	59	13.00
	Freelance	107	24.00
	Operator	7	1.00
	Manager	23	5.00
	Retired	31	7.00
	Other	37	8.00
Annual household income (EUR)	<20,000	130	29.00
	20,000–40,000	263	58.00
	40,000–60,000	54	12.00
	>60,000	6	1.00

**Table 10:** Multinomial logit model (MNL) results.

MNL	Coefficient	Standard Error	|z| > Z *	Confidence Interval	
Price	−0.05442 ***	0.00488	0	−0.06398	−0.04485
Certification	1.49969 ***	0.05833	0	1.38537	1.61402
Place: Restaurant	0.5442 ***	0.05656	0	0.19373	0.41543
ASC Sea urchin	0.0756	0.04718	0.1091	−0.01689	0.16808
ASC Type of dish	−0.00331	0.7800	−0.04	−0.15619	0.14957
ASC Opt-out (no choice)	−0.14356	0.11554	0.214	−0.37	0.08289

Note: ***, * ==> Significance at 1%, 10% level.

**Table 11:** Multinomial logit (MNL) model and random parameter logit (RPL) model parameters.

	MNL	RPL
Log likelihood function	−3488.10	−3165.88
McFadden pseudo R^2^	0.12	0.20
AIC/N	1.93	1.76
Number of respondents	453	453
Number of observations	3624	3624
Number of Halton draws	-	200

**Table 12:** Random parameter logit (RPL) results.

RPL	Coefficient	Standard Error	|z| > Z *	Confidence Interval	
Fixed parameter					
Price	−0.06261 ***	0.00513	0	−0.07267	−0.05256
Certification	1.64471 ***	0.06355	0	1.52017	1.76926
Place of consumption: Restaurant	0.27141 ***	0.05948	0	0.15483	0.38799
ASC Sea urchin	0.06149	0.04811	0.2012	−0.0328	0.15579
ASC Dish type	−0.00494	0.08647	−0.06	−0.17441	0.16453
Random parameter					
ASC Opt-out	−3.14714 ***	0.63295	0	−4.3877	−1.90657
Heterogeneity in mean Parameter variable					
Frequency middle	−0.00777	0.181	0.9658	−0.36251	0.34698
Frequency high	0.15326	0.25238	0.5437	−0.34141	0.64792
Place of purchase: Street vendor	−3.08949 ***	0.80548	0.0001	−4.6682	−1.51078
Place of purchase: Mall	−0.23139	0.93367	0.8043	−2.06134	1.59857
Place of purchase: Kiosk	2.0313 ***	0.50354	0.0001	1.04437	3.01822
Place of purchase: Fish shop	0.63973 ***	0.19944	0.0013	0.24883	1.03063
Place of consumption: sea	−3.03898 ***	0.4782	0	−3.97623	−2.10172
Place of consumption: Home	−0.19562	0.37213	0.5991	−0.92498	0.53374
Place of consumption: Restaurant	−0.17706	0.37275	0.6348	−0.90764	0.55352
Place of consumption: Restaurant-fish shop	−0.18386	0.41752	0.6597	−1.00217	0.63446
Pay attention to price	1.04603 ***	0.15352	0	0.74513	1.34693
Pay attention: Appearance	0.75561 ***	0.14991	0	0.46178	1.04943
Pay attention: Way of conservation	−0.43484 ***	0.16871	0.01	−0.7655	−0.10418
Pay attention: Seller reliability	0.33725 **	0.17028	0.0476	0.00352	0.67099
Pay attention: Place of purchase	−0.01148	0.15103	0.9394	−0.30749	0.28453
Pay attention: Quality label	−0.28709	0.22558	0.2031	−0.72921	0.15503
Influence on price: Appearance	−0.42951 **	0.18299	0.0189	−0.78816	−0.07085
Influence on price: Way of conservation	0.20535	0.15415	0.1828	−0.09678	0.50748
Influence on price: Seller reliability	0.17404	0.15222	0.2529	−0.1243	0.47239
Influence on price: Place of purchase	0.7173 ***	0.17799	0.0001	0.36844	1.06616
Influence on price: Quality label	−0.89667 ***	0.1366	0	−1.1644	−0.62894
Influence on price: Fishing zone	−0.4305 ***	0.15728	0.0062	−0.73876	−0.12225
Behavior: More quantity if price is low	−0.82739 ***	0.2442	0.0007	−1.30601	−0.34876
Behavior: Pay more for a certified product	0.50843 ***	0.18228	0.0053	0.15117	0.86569
Male buyer	−0.90118 ***	0.13998	0	−1.17554	−0.62682
Age	0.04389 ***	0.00628	0	0.03158	0.0562
Years of study	0.05404 ***	0.01985	0.0065	0.01513	0.09294
Income: Medium	−0.31998 *	0.16895	0.0582	−0.65112	0.01116
Income: High	−0.81786 ***	0.22237	0.0002	−1.25371	−0.38202
	Distribution of RPs	SD			
No ASC Opt-out	0.5327 ***	0.07891	0	0.37804	0.68736

Note: ***, **, * ==> Significance at 1%, 5%, 10% level.

## Data Availability

Data are available from the corresponding author.

## Supplementary Materials

The following supporting information can be download at: https://www.mdpi.com/article/10.3390/foods12020418/s1, Table S1: Most attributes used by scientists to elicit consumers’ preferences of sea food products.

## Appendix A. “TuGePlAl” Apulia Project in Brief

Over the last 10 years, the Apulian fishing sector has shown a decisive and systematic decline, caused by the reduction in fishing efforts, both in terms of activity and capacity. The economic and environmental sustainability of the Apulian fishing fleet has been gradually reduced, highlighting the risks of a loss of economic efficiency in the sector and a growing impact on the maintenance of biodiversity. The project proposal is, therefore, the link between responsible fishermen and consumers attentive to sustainable nutrition and respectful of endangered species and the most exploited fish stocks. As such, the project aims to study and build a set of good sustainable fishing practices to be adopted and replicated in other territories. It is intended to promote the development of a system of protection, management, and enhancement of the stock of *Paracentrotus lividus* and *Arbacia lixula* in the “Regional Park of the Coastal Dunes” from Savelletri to Torre San Leonardo, increasing/maximizing, at the same time, the competitiveness and profitability in the regional fishing sector of reference. More details concerning this project and the survey questionnaire may be gathered from the following link: https://www.tugeplal.it/ (accessed on 10 January 2023).
